# IgG Subclasses and Allotypes: From Structure to Effector Functions

**DOI:** 10.3389/fimmu.2014.00520

**Published:** 2014-10-20

**Authors:** Gestur Vidarsson, Gillian Dekkers, Theo Rispens

**Affiliations:** ^1^Department of Experimental Immunohematology, Sanquin Research, and Landsteiner Laboratory, Academic Medical Center, University of Amsterdam, Amsterdam, Netherlands; ^2^Department of Immunopathology, Sanquin Research, and Landsteiner Laboratory, Academic Medical Center, University of Amsterdam, Amsterdam, Netherlands

**Keywords:** immunoglobulin G, IgG, Fc receptors, neonatal Fc receptor, glycosylation, polymorphism, genetic

## Abstract

Of the five immunoglobulin isotypes, immunoglobulin G (IgG) is most abundant in human serum. The four subclasses, IgG1, IgG2, IgG3, and IgG4, which are highly conserved, differ in their constant region, particularly in their hinges and upper CH2 domains. These regions are involved in binding to both IgG-Fc receptors (FcγR) and C1q. As a result, the different subclasses have different effector functions, both in terms of triggering FcγR-expressing cells, resulting in phagocytosis or antibody-dependent cell-mediated cytotoxicity, and activating complement. The Fc-regions also contain a binding epitope for the neonatal Fc receptor (FcRn), responsible for the extended half-life, placental transport, and bidirectional transport of IgG to mucosal surfaces. However, FcRn is also expressed in myeloid cells, where it participates in both phagocytosis and antigen presentation together with classical FcγR and complement. How these properties, IgG-polymorphisms and post-translational modification of the antibodies in the form of glycosylation, affect IgG-function will be the focus of the current review.

## Introduction

Immunoglobulin G (IgG) is one of the most abundant proteins in human serum, accounting for about 10–20% of plasma protein. It is the major class of the five classes of immunoglobulins in human beings, IgM, IgD, IgG, IgA, and IgE. These closely related glycoproteins, composed of 82–96% protein and 4–18% carbohydrate, differ in heavy chain structure and have different effector functions. IgG can be further divided in four subclasses, named, in order of decreasing abundance IgG1, IgG2, IgG3, and IgG4 (1). The subclasses of IgG were discovered in the 1960s following extensive studies using specific rabbit antisera against human IgG myeloma proteins (1). Differences in structure and function of IgG subclasses are summarized in Table 1. Although they are more than 90% identical on the amino acid level, each subclass has a unique profile with respect to antigen binding, immune complex formation, complement activation, triggering of effector cells, half-life, and placental transport.

**Table 1:** **Properties of human IgG subclasses**.

	**IgG1**		**IgG2**		**IgG3**		**IgG4**	
**General**								
Molecular mass (kD)	146		146		170		146	
Amino acids in hinge region	15		12		62^a^		12	
Inter-heavy chain disulfide bonds	2		4^b^		11^a^		2	
Mean adult serum level (g/l)	6.98		3.8		0.51		0.56	
Relative abundance (%)	60		32		4		4	
Half-life (days)	21		21		7/∼21^a^		21	
Placental transfer	++++		++		++/++++^a^		+++	
**Antibody response to**:								
Proteins	++		+/−		++		++^e^	
Polysaccharides	+		+++		+/−		+/−	
Allergens	+		(−)		(−)		++	
**Complement activation**								
C1q binding	++		+		+++		−	
**Fc receptors**								
FcγRI	+++^c^	65^d^	−	−	++++	61	++	34
FcγRIIa_H131_	+++	5.2	++	0.45	++++	0.89	++	0.17
FcγRIIa_R131_	+++	3.5	+	0.10	++++	0.91	++	0.21
FcγRIIb/c	+	0.12	−	0.02	++	0.17	+	0.20
FcγRIIIa_F158_	++	1.2	−	0.03	++++	7.7	−	0.20
FcγRIIIa_V158_	+++	2.0	+	0.07	++++	9.8	++	0.25
FcγRIIIb	+++	0.2	−	−	++++	1.1	−	−
FcRn (at pH < 6.5)	+++		+++		++/+++^a^		+++	

*^a^Depends on allotype*.

*^b^For A/A isomer*.

*^c^Multivalent binding to transfected cells. Adapted from Bruhns et al. (2)*.

*^d^Association constant (×10^6^ M^−1^) for monovalent binding (2)*.

*^e^After repeated encounters with protein antigens, often allergens*.

In addition, IgG antibody responses to different types of antigens leads to marked skewing toward one of the subclasses. Selective subclass deficiencies are usually not detrimental to the individual, but do sometimes lead to enhanced susceptibility toward specific classes of pathogens. This can be caused by complete isotype- or subclass deficiency due to deletions in the Ig loci of chromosome 14, but this is rare (3). More often, one or more of the IgG subclass levels – predominantly IgG2 and/or IgG4 – are below the normal range in healthy individuals (4), which sometimes leads to an impaired response to infections with encapsulated bacteria as will be discussed below. All in all, the acquired variability within the Ig locus seems to have selected for beneficial changes during evolution for optimizing or fine tuning the antibody-mediated immune response.

### IgG antibody responses

The route by which an antigen enters our body and its chemical composition steers the (secondary) immune reaction into preferential patterns of class switching. Besides direct B-cell triggering by the antigen itself, a number of secondary signals will influence differentiation of the B-cell, including recognition by pattern-recognition receptors like Toll-like receptors and cytokines produced by other lymphocytes and antigen-presenting cells (5, 6). For example, protein antigens usually trigger B-cells receiving T-cell help through MHC-class II expressed by the B-cell. For those antigens, class switching tends to be IgG1 or IgG3, but can also be IgG4 or IgE. On the other hand, in the absence of T-cell help, polysaccharide antigens may induce class switching to IgG2 in particular. B-cells undergoing class switching in a primary or secondary immune reaction can also go through subsequent class switching (7), but those events are limited by the availability of remaining heavy chain genes, not excised from the genome in previous class-switching events. The relatively terminal position of the Cγ4 cassette may be one of the reasons why IgG4 responses tend to occur after repeated antigen exposure (8).

#### IgG1

Antibody responses to soluble protein antigens and membrane proteins primarily induce IgG1, but are accompanied with lower levels of the other subclasses, mostly IgG3 IgG4 (9). Because IgG1 is normally the most abundant subclass, a lack of IgG1 seen in a variety of primary and secondary antibody deficiencies, can result in decreased total IgG levels (hypogammaglobulinemia). IgG1 deficiencies, sometimes in combination with other IgG subclass deficiencies, are associated with recurrent infections (10).

#### IgG2

Immunoglobulin G-antibody responses to bacterial capsular polysaccharide antigens can be almost completely restricted to IgG2 (9, 11–13), and IgG2 deficiency may result in the virtual absence of IgG anti-carbohydrate antibodies (14), although these responses can also be compensated for by enhanced levels of other IgG subclasses, in particularly by elevated IgG1 and IgG3 levels (15). An increased susceptibility to certain bacterial infections is associated with IgG2 deficiency, suggesting a role of IgG2 in the defense to these pathogens (16). Low concentrations of IgG2 often occur in association with a deficiency in IgG4 and/or IgA1 and IgA2 (17).

An extensive analysis of anti-carbohydrate reactivities in intravenous immunoglobulin revealed that although IgG2 indeed represents the bulk of the reactivity to many glycans, this is not always the case (18). IgG1 antibodies have also been reported to prevail against *Haemophilus influenzae* b polysaccharide during natural infections (9). In normal immune responses in healthy individuals, IgG1 and IgG3 responses can also be observed, and certainly against protein-conjugated glycans, which happens in the reaction to second-generation pneumococcal vaccines (19).

#### IgG3

IgG3 antibodies are particularly effective in the induction of effector functions. Being a potent pro-inflammatory antibody, its shorter half-life may function to limit the potential of excessive inflammatory responses. However, the finding that some individuals bearing the G3m allotypic “s” or “15” marker [i.e., G3m (s)/G3m (15) and G3m (st)/G3m (15, 16) allotypes] also have IgG3 with prolonged half-life may challenge that assumption (20). Curiously, IgG3 levels in these individuals do not seem to be increased, which may be explained by γ3-promotor polymorphisms known to affect the frequency of class switching to IgG3 in G3m (g) allotypes, explaining the low concentration in most G3m (g) homozygous individuals (21, 22). Viral infections in general lead to IgG antibodies of the IgG1 and IgG3 subclasses, with IgG3 antibodies appearing first in the course of the infection (9). IgG3-dominated responses appear to be rare. A curious example is the so-called anti-hinge antibodies (23), which bind to the hinge region of Fab2 fragments but not intact IgG antibodies. Also, antibodies to P and P^k^ blood group antigens are largely restricted to IgG3 (24). Responses against other red cell antigens (e.g., RhD) and platelets (e.g., human platelet antigen 1a), as seen in transfusion and in pregnancies, are often dominated by IgG1, IgG3, or both (25–27). Decreased IgG3 levels are frequently associated with other IgG subclass deficiencies (28).

#### IgG4

Allergens are often good inducers of IgG1 and IgG4, in addition to IgE. IgG4 antibodies are often formed following repeated or long-term exposure to antigen in a non-infectious setting and may become the dominant subclass. Examples are long-term bee keepers and allergic individuals that underwent immune therapy (8, 29–31). In immunotherapy, relief of symptoms appears to correlate with IgG4 induction. Switching to IgG4 may be modulated by IL10, linking this subclass downregulation of immune responses or tolerance induction (8, 32). IgG4 may also represent the dominant antibody subclass in immune responses to therapeutic proteins, such as factor VIII and IX (33–35) and at least some recombinant antibodies such as adalimumab (36). Furthermore, helminth or filarial parasite infections may result in the formation of IgG4 antibodies (37, 38), and high IgG4 titers can be associated with an asymptomatic infection (39).

Isolated IgG4 deficiencies are rare; it is uncertain what the possible consequences are. On the other hand, a group of disorders, nowadays referred to as IgG4-related diseases (IgG4RD), are characterized by elevated serum IgG4 concentration and tissue infiltration by IgG4-positive plasma cells and may affect a number of organs (40, 41). The spectrum of IgG4RD is wide and includes patients with autoimmune pancreatitis (AIP), Mikulicz’s disease, hypophysitis, Riedel thyroiditis, interstitial pneumonitis, interstitial nephritis, prostatitis, lymphadenopathy, retroperitoneal fibrosis, inflammatory aortic aneurysm, and inflammatory pseudotumor (42). In AIP patients, elevated serum IgG4 (>1.4 g/L) is observed in 70–80% of the cases, as well as in 10% of pancreatic cancer patients. However, as 5% of the normal population also has elevated IgG4 levels, this makes it only suitable for diagnosis in combination with other features of AIP.

## Structure

**Figure 1:**
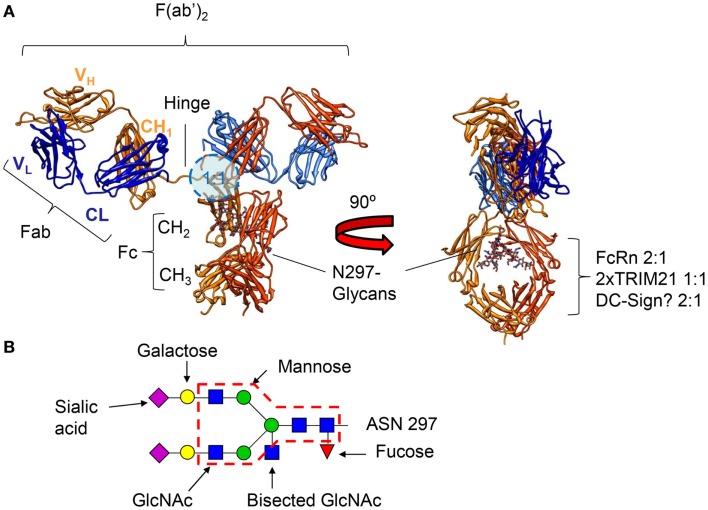
**(A)** Crystal structure of an human IgG1 molecule (1HZH) viewed from two different angles, demonstrating the flexibility of the two Fab fragments with respect to each other and the Fc tail. The binding location for FcγR, binding IgG asymmetrically in a 1:1 configuration (46–49), is indicated by the blue circle (lower hinge, upper CH2) on the left, and the location of the binding motifs for FcRn, TRIM21, and the potential site for binding of DC-SIGN on the right (intersection of CH2 and CH3). FcRn, and the potential binding site of DC-SIGN bind IgG in a 2:1 configuration (50–52), respectively, while a dimer of TRIM21 binds IgG in a 1:1 configuration (53). The N-linked glycan at position 297 attached to each of the heavy chains is shown on the right. **(B)** The N-linked glycan found at position 297 can be found as a core structure, common to all IgG found in human beings and rodents (core structure indicated with a red dashed line), but can be found with either an addition of fucose, bisecting *N*-acetylglucosamine (GlcNAc), one or two galactose, and one or two sialic acid residues.

**Figure 2:**
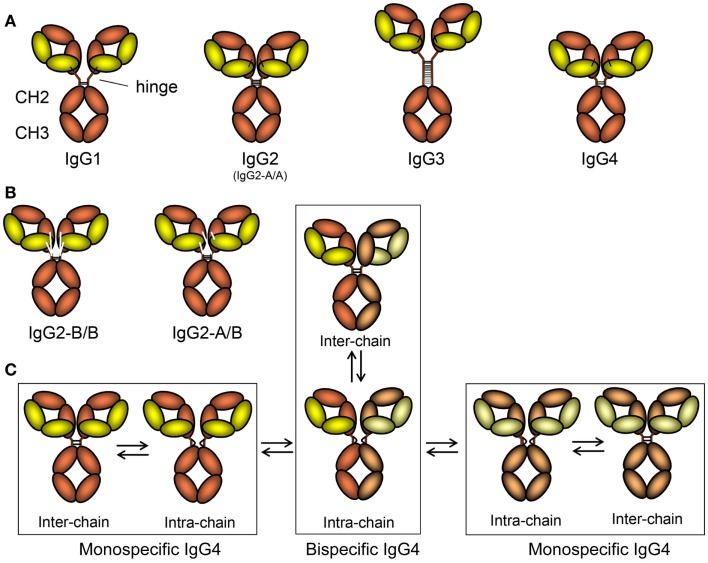
**The schematic layout of the IgG subclasses and isomers thereof**. **(A)** The IgG subclasses, indicating how the different heavy and light chains are linked, the length of the hinge, and the number of disulfide bridges connecting the two heavy chains. For orientation, and comparison with Figure 1, the location of the hinge, CH2, and CH3 domains are shown. The classical A/A isoform of IgG2 with four different disulfide bridges between the two heavy chains is depicted here, but in **(B)** the B/B form, with only two disulfide bridges and alternative linkages of the light chain to the heavy chain form is shown, together with the intermediate A/B form. **(C)** Isomers of IgG4 resulting in half-molecule exchange. On the far left and far right, two classically depicted IgG4 clones in slightly different colors are shown just after secretion from B-cells. These are connected with two inter-chain disulfide bridges. However, these are in fact in equilibrium where these are reduced creating forms without covalent linkages between the symmetric molecules. This form can either revert back to covalently linked form or swap heavy chains in a stochastic process with that of neighboring IgG4 molecule creating a asymmetric bispecific IgG4 (bottom middle) that is also in flux, reverting into covalently linked IgG4 (top, middle). By this process, most IgG4 found in human beings (expressing the IgG4-R409 allotype, see text for more details and Figure 3) are monovalent-bispecific molecules.

Similar to the other isotypes, the IgG immunoglobulin molecule consists of four polypeptide chains, composed of two identical 50 kDa γ heavy (H) chains and two identical 25 kDa κ or λ light (L) chains, linked together by inter-chain disulfide bonds. Each heavy chain consists of an N-terminal variable domain (VH) and three constant domains (CH1, CH2, CH3), with an additional “hinge region” between CH1 and CH2 (Figure 1A). Similarly, the light chains consist of an N-terminal variable domain (VL) and a constant domain (CL). The light chain associates with the VH and CH1 domains to form a Fab arm (“Fab” = fragment antigen binding), and functionally, the V regions interact to form the in antigen-binding region – acquired through differential assembly of Variable, Diversity (VH only), and Joining gene segments and inclusion of somatic mutations (43–45), although their relative contribution to antigen binding varies greatly. Two heavy chain–light chain heterodimers (HL) combine into a single antibody molecule (H_2_L_2_) via disulfide bonds in the hinge region and non-covalent interactions between the CH3 domains (Figure 2A). The part of the antibody formed by the lower hinge region and the CH2/CH3 domains is called “Fc” (“fragment crystalline”).

**Figure 3:**
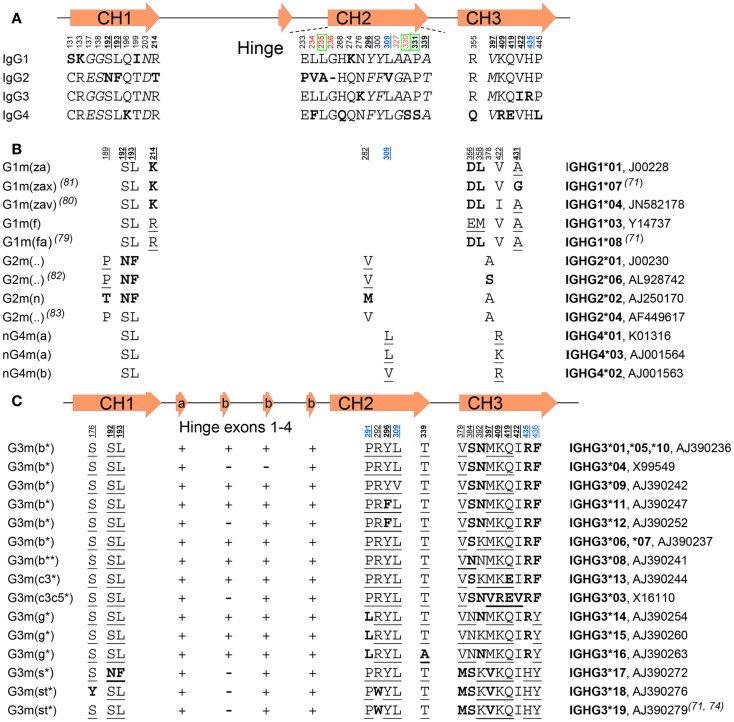
**IgG subclasses and IgG allotypes**. **(A)** All differences between the IgG isotypes depicted schematically according to their localization (numbered below a graphical representation of the gene) in the different domains and exons depicted above the sequence. Bold underlined numbers (EU numbering) contain isoallotypic variant at that position. Amino acids depicted in bold varies from the other subclasses, but amino acids depicted in italics are present in two subclasses. The green boxed amino acids numbers are residues involved in binding to C1q, in red amino acids involved in FcγR binding, and in blue residues involved in binding to FcRn. “-“ instead of a letter for amino acid stands for the missing G236 residue in IgG2. **(B)** The amino acid variation found within IgG1, IgG2, and IgG4 allotypes, and **(C)** among the IgG3 allotypes. The presence or absence of the two kinds of IgG3-hinge exons (a, and b) are indicated by “+” or “–” in **(C)**. For **(B,C)**, amino acids in bold are those unique for subclass or allotype, and those underlined isoallotypes, as this amino acid is also found in other subclasses at this position. The unique IMGT numberings and a representative sequence accession number are indicated on the right. For some of the allotypes, the IMGT numberings are represented by several different genes, but encoding for identical hypothetical proteins.

The global structures of the four human IgG subclasses are very similar (Figures 1 and 2A), but with important differences between each subclass that affect their binding to accessary molecules and receptors, affecting their functionality (Table 1). The four subclasses show over 90% homology in amino acid sequence, with differences that are not randomly distributed. Much variation is found in the hinge region and N-terminal CH2 domain, whereas fewer amino acid differences are found in the other domains. Of these, least is known about the functional consequences – if any – of structural variations found within the CH1 domain. On the other hand, structural differences in the CH2/CH3 domains, forming the Fc tail, are relatively well studied.

The residues most proximal to the hinge region in the CH2 domain of the Fc part are responsible for effector functions of antibodies as it contains a largely overlapping binding site for C1q (complement) and IgG-Fc receptors (FcγR) on effector cells of the innate immune system.

A highly conserved N-linked glycosylation site at position 297 is located at the interface between the two CH2/CH3 forming the Fc of an IgG molecule (Figure 1A) that is both responsible for subtle but important changes of quaternary structure of the Fc – allowing for a more exposed docking-site for FcγR. As discussed further below, these glycans also directly participate in the FcγR binding, but can also modulate these interactions through highly specific modifications of the N297 glycan – changes that seem to be regulated during specific immune responses in human beings (54, 55). Although this glycosylation site is often regarded as the only glycosylation site in IgG, the V region of approximately 10–15% of all antibodies is also glycosylated. These sites most often arise through VDJ-recombination or somatic hypermutation (43), and this glycosylation has been reported to affect antigen-binding characteristics (56–59) and allowing binding to regulatory lectins. This, in turn, can also modulate the activation-threshold required for B-cell stimulation, and has been described as a positive selection signal in certain types of follicular lymphomas (60).

The interface between the CH2–CH3 domains also contains the binding site for the neonatal Fc receptor (FcRn), responsible for the prolonged half-life of IgG, placental passage, and transport of IgG to and from mucosal surfaces. Little variation exists in this region, with FcRn binding only minimally affected, except perhaps for IgG3 as discussed further below.

However, the binding profiles of FcγR and C1q to the different IgG subclasses go hand in hand (Table 1); each IgG subclass having its distinctive pattern that has been investigated in detail. How the differences in the primary sequences of the IgG subclasses (Fc and hinge) lead to variations in tertiary structural elements, thereby critically influence the properties of each subclass, is the subject of the following sections.

### Structural Variation in the Hinge Region

The hinge region forms a flexible linker between the Fab arms and the Fc part. Length and flexibility of the hinge region varies extensively among the IgG subclasses (Figure 2) (61). This affects the possible conformations of the Fab arms relative to the Fc domain as well as to each other. The hinge exon of IgG1 encompasses 15 amino acids and is very flexible. IgG2 has a shorter hinge than IgG1, with 12 amino acid residues. The lower hinge region of IgG2 (actually encoded by the CH2 region) also has a one amino acid deletion (lacking one of the double Glycines found at position 235-6), resulting in IgG2 having the shortest hinge of all the IgG subclasses. In addition, the hinges of IgG2 are even more rigid due to a poly-proline helix, stabilized by up to four (with some exceptions discussed below) extra inter-heavy chain disulfide bridges (Figure 2). These properties restrict the flexibility of the IgG2 molecule. Similarly, the hinge region of IgG4 also contains 12 amino acids and is thus shorter than that of IgG1. Its flexibility is intermediate between that of IgG1 and IgG2 (62). Unlike IgG2, it does encode for the CH2-encodied glycines 235-6 in the lower hinge (Figure 3A).

IgG3 has a much longer hinge region than any other IgG subclasses or Ig human isotype, i.e., about four times as long as the IgG1 hinge, containing up to 62 amino acids (including 21 prolines and 11 cysteines), forming a poly-proline helix with limited flexibility (63, 64). The exact length of the hinge varies between allotypes of IgG3, which apparently has undergone much more evolutionary radiation than the other subclasses (Figures 3B,C) as discussed below. In IgG3, the Fab fragments are relatively far away from the Fc fragment, giving the molecule a greater flexibility. This long hinge of IgG3 is a result of duplications of a hinge exon, encoded by one exon in IgG1, IgG2, and IgG4, but up to four exons in IgG3. One of those exons is common to all IgG3 allotypes, but it also has 1–3 copies of a homologous second type of IgG3-hinge exon (Figure 3C). The elongated hinge in IgG3 is also responsible for its higher molecular weight compared to the other subclasses. The difference in hinge flexibility influences the relative orientation and movement of the Fab arms and Fc tail of the IgG antibody.

Binding sites for C1q and/or FcγR may be partially or completely shielded by Fab arms, affecting binding of the IgG to these molecules. The relative flexibility of the Fab arms with respect to the Fc differs between subclasses as follows: lgG3 > lgG1 > lgG4 > lgG2 (62, 65), which also reflects the relative binding of these subclasses to FcγR and C1q, although this only partially explains the respective activities of the IgG subclass, as discussed elsewhere in this review. This flexibility also affects antigen-binding capacity and immune complex formation.

### Inter-chain disulfide bonds

The four IgG subclasses also differ with respect to the number of inter-heavy chain disulfide bonds in the hinge region (Table 1; Figure 2A). In addition, both IgG2 and IgG4 are found as several isomers, in which the hinge disulfide bonds are differentially inter-connected (see below). Another structural difference between the human IgG subclasses is the linkage of the heavy and light chain by a disulfide bond. This bond links the carboxy-terminal cysteine of the light chain to the cysteine at position 220 (in IgG1) or at position 131 (in IgG2, IgG3, and IgG4) in the CH1 domain (Figure 2A). These two positions are spatially juxtaposed and the essential structure and function of the molecule appears to be conserved between the two types of linkage between heavy and light chain.

### Hinge isomers in IgG2 and IgG4

In IgG2, structural hinge isomers have been observed as a result of alternative formation of disulfide bonds between the cysteines in the hinge region of the heavy chains and those involved in the formation of disulfide bonds between the light and heavy chain (Figure 2B) (66, 67). These isomers were found particularly in IgG2 antibodies with kappa-light chains, but much less for lambda light chains. The major forms are the classical A form, with four disulfide bridges between the two IgG2 heavy chains, and the B form in which one hinge cysteine forms a disulfide bond with the light chain. However, other configurations exist (67, 68), as these isoforms apparently form independent of each other, giving rise to A/A, B/B, but also A/B isoforms (Figures 2A,B). FcRn binding does not seem to be different for the different isomers (69). IgG2 has also been reported to form covalent dimers (70), which might be regarded as an additional isomer.

Two isomers of IgG4 differing in the disulfide bonding of hinge cysteines coexist. The core hinge of IgG is formed by a CXXC motif, also found in redox-reactive proteins such as thioredoxins (71). Compared to IgG1, with a relatively rigid CPPC motif (72), intra-chain disulfide bonds are more easily formed between these cysteines found at positions 226 and 229 in IgG4, which possesses a CPSC core hinge (Figure 2C). The result is an observable amount of non-covalently linked half-molecules (consisting of one heavy and one light chain, HL, as opposed to the classical configuration of H_2_L_2_) in addition to covalently linked inter-chain isomers (Figure 2C) (73, 74). An S228P mutant of IgG4, thus with an IgG1-core hinge, does not form half-molecules, which is in agreement with the finding that this species does not occur in IgG1. The process is reversible but depends on redox conditions. Formation of the intra-chain isomer (half-molecules) is an important step in the “Fab arm exchange.”

### IgG4 Fab arm exchange

*In vivo*, half-molecules of IgG4 recombine randomly with other half-molecules of IgG4, combining specificities of two IgG4 molecules, effectively resulting in monovalent-bispecific antibodies (Figure 2C) (75, 76), and is controlled by redox conditions (74, 77). The unique S228 in the core hinge of IgG4 allows formation of the intra-chain isomer, and R409 (rather than the equivalent lysine in IgG1) results in weaker CH3–CH3 interactions (Figure 3A) (77, 78). Both determinants appear to be required to observe Fab arm exchange *in vivo* (74) and have been observed for the therapeutic IgG4 antibody natalizumab (79). The functional consequence of this are at least twofold. The resulting IgG4 antibody cannot effectively crosslink the target antigen. Furthermore, multivalent target binding is not possible for bispecific antibodies, resulting in a lower avidity, although the affinities of IgG4 antibodies are generally high. In combination with the observations that IgG4 responses seem to dominate after repeated antigen exposure (e.g., bee venom), and because IgG4 has low affinity to activating FcγR while retaining relatively high affinity to the inhibiting FcγRIIb (Table 1), this may serve as an evolutionary way to prevent excessive immune responses against these sterile antigens not posing infectious threats. IgG4 has, therefore, been characterized as “blocking antibody,” especially in the context of allergy, where it may compete with IgE for allergen binding (8).

## Allotypes

In addition to isotypic variation, allelic variation is found among the IgG subclasses (Figure 3) (80). These polymorphic epitopes of immunoglobulins that can differ between individuals and ethnic groups (81) were originally discovered on the basis of serological findings (82), as immunogenic determinants were found on IgG from some individuals but not others. Subsequently, allotypic variations were genetically analyzed, and a number of structural determinants identified (83–86). A large number of polymorphisms were found in IgG, a finding made useful for example in paternity testing and forensic medicine before HLA typing became available (82). Exposure of an individual to a non-self allotype can induce an anti-allotype response, and may occur in transfused individuals (82) and has even been described in a pregnant woman (87). However, not all variations in IgG amino acid sequence lead to determinants that are immunogenic because some determinants are found in other isotypes, and are therefore called isoallotypic variants (Figure 3). Other variations in amino acid sequence can be present at sites that are minimally exposed and, therefore, may not result in determinants that can be serologically discriminated. Therefore, the original alphabetical and numerical designation systems (Gm or Genetic marker, including a subclass designation, e.g., G1m for IgG1 (81), based on serology, do not fully cover the structural variation among different allelic forms of IgG subclasses. This is particularly true for IgG3, which shows extensive polymorphisms – often of isoallotypic origin (Figure 3C) (83). An example is the non-a or nG1m (a) determinant (E and M at positions 356 and 358, respectively) that can also be found on IgG2, 3, and 4 (81). Therefore, in order to refer uniquely to a particular polymorphic variant, it is advisable to use the IMGT allele names rather than either the alphabetical or numerical allotype nomenclature (Figure 3).

The main allelic forms for IgG1 (Figure 3B) are G1m (z,a), G1m (f), and G1m (f,a) (81, 88). The G1m (f) allele is only found in Caucasians, whereas the G1m (f,a) allele is common in Orientals, but other variants, G1m (z,a,x) and G1m (z,a,v), have also been described (89, 90). Several allelic forms of IgG2 are known, including G2m (..) variants, which, together with IgG4, serve as examples of IgG variants without actual allotypic determinants, as here the amino acids present at position 189 and 282 are isoallotypic and also found in the other subclasses (Figures 3A,B) (91, 92). For IgG3, many allelic forms are known, with the most important ones encoding for amino acid changes shown in Figure 3C.

Since some allotypes have proven to be immunogenic, they may be relevant to consider when developing therapeutic antibodies. Treatment using therapeutic monoclonal antibodies can in principle also lead to an anti-allotype response. However, to date, little evidence has been found for significant anti-allotype responses, e.g., adalimumab (93) or infliximab (94). There are no known allotypic variations that result in a functionally different antibody, except for IgG3, where a few isoallotypic variants result in extended half-life (discussed in Section “FcRn”). Furthermore, several polymorphisms in the CH3 domain affect the CH3–CH3 interdomain interactions, which for different IgG3 isoallotypic variants result in these interactions to vary multiple orders of magnitude, which potentially has consequences for e.g., C1q binding (77). Interestingly, plasma IgG concentrations of an individual appear to correlate with Gm allotype (21, 28, 95), which may be related to the actual single-nucleotide polymorphisms (SNP) causing the allotypes themselves, or silent SNP, that in turn may affect unfavorable codon-usage or alter secondary mRNA structures affecting transcription (96–98). In addition, the allotype may also affect class-switching efficiency and thereby serum concentrations, through variations within the non-coding switch regions inherited haplotype. This is because these sterile promoters undergo transcription preceding actual class switching (21, 22, 98), and allotype differences found within these region affect class-switching efficiency directly, explaining the lower levels found of IgG3 in g3m (g) individuals compared to G3m (b).

For IgG4 no serologically important allotypes are known to exist, but isoallotypes have been described (81). Furthermore, a polymorphic variant exists (K409 instead of R409) that is unable to participate in Fab arm exchange (84). It is unknown if carrying this variant has any biological consequences (e.g., because this kind of IgG4 can potentially crosslink antigens as opposed to the classical monovalent IgG4 type), as the apparent low allelic frequency makes homozygotes rare, at least in the western-European population.

In conclusion, most of the genetic variation in IgG have potential implications far beyond the original serological findings, as non-serologically important variations exceed those by far. As of yet, we still do not fully know the extent of these variability as this has not yet been revisited by modern techniques. This includes on the number of unknown variants, the frequency by which they appear within and between populations. This can have functional consequences, on expression levels (21), half-life, FcγR binding [antibody-dependent cell-mediated cytotoxicity (ADCC), ADCP], tendency to form oligomers (99) and activate complement, and influence on immunogenicity – again within and between populations – and, therefore, have important consequences for antibody-mediated immunotherapies.

## Glycosylation

Immunoglobulin G contains a conserved glycan at position N297 of the heavy chains. In addition, roughly 10–20% of the Fab have N-glycosylation sites in the binding region. The core structure of the IgG glycans comprises *N*-acetylglucosamine (GlcNAc) and mannose residues (Figure 1B). This can be further extended with galacose, sialic acid, core fucosylation, and bi-sected GlcNAc (Figure 1B). Several dozen IgG-Fc glycoforms have been found in healthy human serum, of which only a handful represent the dominant form (fucosylated species in one of the following configuration: without galactose, with one- or two galactose residues, or with two galactose and a single sialic acid residue, Figure 1B) (54, 100, 101). Between Fab and Fc glycans, there are several differences in glycosylation, those most pronounced being markedly increased levels of bisection, galactosylation, and sialylation in the Fab glycans (including di-sialylation hardly seen in the Fc), but reduced fucosylation (~94% for Fc, vs ~70% for Fab) (100, 102). This is partially due to accessibility for glycosyltransferases and glycosidases, Fab sites are generally more accessible compared to the conserved site in the Fc, which lies more buried between the two heavy chains (Figure 1A). In addition, glycosylation levels are also controlled by availability of glycosyltransferases in the B-cells. In several health and disease settings, a shift toward certain Fab- and Fc-glycoforms of antibodies has been reported (54, 55, 103). This shift may occur through epigenetic influence on expression of glycosyltransferases (104), which is clearly affected by various factors including, age, pregnancy, hormones, cytokines, bacterial DNA, and food metabolites (105–107), although the complete working mechanism of regulation of expression in B-cells has to be determined.

As mentioned above, the composition of the N297 glycan influences the quaternary structure of the Fc. Interactions of the glycan with the protein backbone stabilize the Fc (108). Addition of the Fc-glycan gives the IgG-Fc a more open conformation, allowing binding to FcγR (109, 110). In addition, the glycan is in close proximity to the FcγR itself, contribution to the binding through glycan protein binding (110). However, perhaps even more important is the fact that human FcγRIIIa and FcγRIIIb express a conserved glycan at position 162, entering the Fc-space confining the Fc-glycan, enabling a direct glycan–glycan interaction (46, 111).

Our knowledge on how the different glycoforms affect the effector function of IgG is still in its infancy, but some aspect have been becoming increasingly clear as summarized below.

### Fucosylation

It has been known for quite some time that core fucosylation of the IgG-Fc affects binding to FcγRIIIa (112), with non-fucosylated antibodies binding FcγRIIIa much stronger. This higher affinity translates into higher ADCC and phagocytosis of targets by these antibodies (54, 112), and has been put to use in therapeutic antibodies, like rituximab, to increase the efficacy of treatment [reviewed by Ref. (113, 114)]. More recently, it has become clear that this also applies to FcγRIIIb expressed on granulocytes (54, 115, 116). The molecular nature of this increased affinity is discussed below (see FcγRIIIa). After vaccination, or apparently after normal immune responses, the IgG responses in human beings are restricted to IgG with core fucose attached, as seen against soluble proteins during influenza or tetanus toxoid vaccination (117). This also reflected by the fact that ~94% of IgG-Fc glycopeptides are fucosylated in total serum. Even more strikingly is the fact that IgG fucosylation is prevented in some immune responses against particulate antigens, e.g., red blood cells and platelets (54, 55, 103), but also witnessed in some elite controllers of HIV (57). Thus apparently, FcγRIII-mediated IgG responses can be fine-tuned through IgG fucosylation toward more pro- or anti-inflammatory effects.

### Bisection

Slight changes in bisection have been detected for some antigen-specific IgG responses (54, 102, 103, 118). Little is known about the importance of the biological implication of these changes. It has been described that fucosylation and bisection occurs in a reciprocal manner, with proximal bisection blocking fucosylation of IgG, making it difficult to discriminate the effect of bisection from core fucosylation (119–121).

### Galactosylation

To our knowledge, no data have been published to date on the influence of galactosylation on the level of antigen-specific IgG. Immunization seems to result in a transient increase in galactosylation of antigen-specific IgG in human beings, while having no effect on total IgG galactosylation (117). However, general decrease in galactosylation has been found in several autoimmune diseases [reviewed in Ref. (122)], suggesting degalactosylated IgG to be more pathogenic, or galactosylated IgG to have anti-inflammatory activity. This includes rheumatoid arthritis, a disease that often goes into remission during pregnancy – correlating with the general increase in galactosylation in pregnancy (123).

### Sialylation

As the terminal – and the only charged – sugar moiety, sialylation has been proposed to have the most effect on the structure of the Fc domain of the antibody, by closing the binding site for activating FcγRs, but opening up a cryptic binding site for DC-SIGN in the CH2-CH3 interface (50). In proof of this, actual comparisons of IgG with or without Fc-sialylation have confirmed this (124), and mouse IgG decreased affinity to mouse FcγR in general (125), although systematic analyzes of the importance of this for human IgG-FcγR binding is lacking. Increased sialylation of IgG generally follows increased galactosylation as galactosylated IgG is the substrate for sialyltransferases (Figure 1B) (54, 103, 126). Binding to DC-SIGN sialylated antibodies has been suggested to have strong immunomodulatory function as described below (see DC-SIGN).

## Effector Mechanisms

### Binding to effector molecules

Antibodies link the adaptive immune system with the effector mechanisms of the innate immune system. They form a bridge by combining antigen-binding sites with binding sites for many innate receptors and adaptor molecules. The effector mechanisms that will be triggered vary between the different immunoglobulin subclasses. Typically, IgG1 and IgG3 are potent triggers of effector mechanisms, whereas IgG2 and IgG4 will induce more subtle responses, and only in certain cases. However, these antibodies remain capable of neutralizing virus particles and toxins. Below, binding to C1q and FcRns is discussed, emphasizing the structural aspects that differ between the subclasses (Table 1).

#### C1q

Upon binding to target surfaces, IgG, as well as IgM, can activate complement. Complement activation is initiated through binding and subsequent activation of C1q, leading to deposition of C3b to further opsonize the target, but also to the formation of the membrane attack complex, C5–C9, causing disruption of the targeted bilipid membrane (127). IgG1 and IgG3 can efficiently trigger this classical route of complement (128), but IgG2 and IgG4 does so much less efficiently or only under certain conditions for IgG2. This is due in large part to the reduced binding of C1q to the latter subclasses (128–130), although it has also been described that in addition to C1q binding, downstream events of the complement cascade (C4b deposition) are differentially affected by the different IgG subclasses (128). Residues in the CH2 region important for C1q binding include L235, D270, K322, P329, and P331 (130–133). In IgG2, reduced C1q binding appears to be largely caused by residue A235 (which is Leu in other subclasses) (132), whereas in IgG4, P331 is – at least in part – responsible for the reduced or absent binding of C1q (130, 133). Structural determinants in the middle or “core” hinge region (residues 226–230) can influence the binding of C1q (134). On the one hand, rigidity in this region contributes favorably to C1q binding, whereas removal of cysteine bonds negatively affects binding. It has also been suggested that the relatively long hinge of IgG3 makes the C1q binding site more accessible resulting in more efficient complement activation (135, 136). However, IgG3 engineered with a short IgG4 hinge binds C1q efficiently, although complement activation was somewhat reduced (137).

Interestingly, IgG has recently been suggested to form hexamers by interactions through the CH2–CH3 interface when opsonized on target surfaces, forming an optimal platform for the hexameric configuration of C1q (99). These data are supported by mutation in this interface, e.g., I235 in the CH2 and H433 in the CH3 that individually affect complement activation through C1q (99).

Curiously, engineered IgG1/3 hybrids with an IgG1-CH1, and hinge regions were found more potent in complement activation compared to wild-type IgG3, with the largest contribution arising from the CH1 domain swap (138). Conversely, the binding of C1q to IgG4 can be influenced by shielding of the potential binding site by Fab arms (74, 136, 139, 140). The orientation of the Fabs have been modeled to be perpendicular to that of the hexameric platform of IgG on solid surfaces and in solution (99) and may thereby affect C1q biding, although this needs to be confirmed. IgG4 also results in less complement activation by forming small immune complexes, probably because of their monovalency, and in this way can even reduce complement activation by IgG1 antibodies (141). Although the short hinge of IgG2 may lead to similar shielding of the potential C1q binding site, a notion that fits with its general poor activation of the classical complement cascade, IgG2 can activate this cascade at high densities of surface antigens, as is the case for polysaccharides – to which IgG2 antibodies tend to form (19, 142, 143). At these high epitope densities, IgG2 may be more likely to efficiently form hexamers, increasing the avidity of this subclass for C1q substantially (99).

## Fcγ-Receptors

FcγR bind to a region partially overlapping the C1q binding site. The binding of IgG to these receptors has been studied in detail. For all FcγR interactions, the stretch of amino acids comprising the N-terminus of the CH2 domains and strands adjacent in the three dimensional immunoglobulin fold are important for binding. In general, this encompasses amino acids 234–239, 265–269, 297–299, and 327–330 (110, 144). However, each of the IgG subclasses has a unique binding profile to each FcγR (Table 1) (2), and their expression profiles are highly variable between different immune cells of myeloid and NK cell origin (145). A major distinction can be made between IgG1/IgG3 that interact efficiently with most FcγR, and IgG2/IgG4, which show reduced affinity to a number of FcγR. Furthermore, monomeric IgG3 binds more efficient than monomeric IgG1 to FcγRIIa, FcγRIIIa, and FcγRIIIb, and binding efficiency of complexed IgG3 to all FcRns exceeds that of IgG1 (2). Structural determinants responsible for the differences between IgG1 and IgG3 are still unknown. Below, we discuss structural differences that are known to be responsible for the subclass-specific variations.

### FcγRI

Although FcγRI is often referred to as a single entity, FcγRI consists of three homologous genes on the short arm of chromosome one (146), and several alternative splice variants have been described (147). However, only the existence of the full-length form, FcγRIa, consisting of three extracellular domains has been studied in detail. The gene encoding for the potential FcγRIb variant, potentially consists of a nearly identical receptor with only the two N-terminal extracellular Ig-domains, but retaining the intracellular cytoplasmic tail, while FcγRIc would also lack the cytoplasmic tail and transmembrane region, and would, therefore, be predicted to represent a secreted form (Figure 3). FcγRIa receptor binds all human IgG subclasses except IgG2, and unlike the other FcγR, contains its unique third membrane-proximal immunoglobulin domain that is also responsible for its higher affinity to IgG (148). Mutations of IgG1 in the lower hinge to the IgG2 equivalents, in particular E233P, L235A, and G236Delta, abrogate binding (149–153). Binding to FcγRI is reduced for IgG4 (2), and both P331S and L234F are implicated to account for the reduced binding in comparison to IgG3 (152), but P331 may not be important for binding of IgG1 (151, 153). An IgG3 with a partially deleted hinge was found to have reduced binding to FcγRI and FcγRIIa (154).

### FcγRIIa

FcγRIIa is the most widely expressed FcγR on myeloid cells and has been described as the only FcγR with significant binding to IgG2 (2, 155–157). Binding is more efficient for the 131H (“low-responder,” LR) variant than the 131R (“high-responder,” HR) variant (nomenclature based on differential binding to mouse IgG1, which binds the HR much better) (155). Binding affinity varies among subclasses as follows: IgG3 > IgG1 > IgG4 = IgG2. Recently, a crystal structure of the complex of IgG1 Fc with FcγRIIa was published (144), and contact residues relating to differences in subclass binding include L234, L235, G236 in the lower hinge, and the structurally adjacent A327. Significantly, the 131R-site in FcγRIIa is also in close proximity to the lower hinge in this co-crystal structure. Thus, the lowered binding affinity of IgG2 to FcγRIIa, and the differential binding to the HR/LR-form of FcγRIIa, may also be attributed to differences in the hinge of IgG2.

### FcγRIIb/IIc

The extracellular domain of the inhibiting FcγRIIb is identical to the activating FcγRIIc that is expressed in ~11% of individuals (158, 159). Binding to the inhibitory receptor FcγRIIb or IIc is weak for all subclasses, generally preferring IgG3 = IgG1 = IgG4 > IgG2. Interestingly, dissociation constants for binding of monomeric IgG1 and IgG3 are similar, but immune complexes of IgG3 seem to bind more efficiently compared to IgG1 (2). Binding to most activating FcRns is lower for IgG4 compared to IgG1, but this is not the case for the inhibitory receptor FcγRIIb. This altered balance between binding to activating receptors in comparison to inhibitory receptors may be an important feature of IgG4 that contributes to its low pro-inflammatory capacity.

### FcγRIIIa

Two allotypic variants of FcγRIIIa exist: F158 and V158. The V158 variant has greater affinity for all subclasses, and for IgG3, binding efficiency approaches that of FcγRIa (2), with general affinities following IgG3 > IgG1 > > IgG4 > IgG2. Besides changing amino acids 233–236 from IgG1 to the IgG2 equivalents, A327G (Ala present in IgG1 and IgG3, Gly in IgG2 and IgG4) also results in decreased binding (151). Binding affinity of FcγRIIIa seems to be particularly sensitive to core fucosylation of the N-linked glycan at N297 of the Fc tail of IgG, as its binding affinity can be enhanced up to 50× times – with corresponding increase in effector function – if the Fc tail is not fucosylated (160, 161). Recent work by Ferrara et al. has pinpointed this interaction to be due to carbohydrate–carbohydrate interactions between the glycan on N297 of the heavy chain and glycosylation of FcγRIIIa at position 162 – a position unique to both FcγRIIIa and FcγRIIIb (46).

### FcγRIIIb

There are also functional allotypic variations of the neutrophil FcγRIIIb, referred to as human neutrophil antigen 1 (NA1/HNA1a) and (NA2/HNA1b) (162). The FcγRIIIb-NA1 form is capable of better ingestion of IgG1- or IgG3-opsonized particles than RIIIb-NA2 (163). FcγRIIIb generally binds IgG1 and IgG3 but not IgG2 and IgG4, with IgG3 binding better than IgG1 (2). A crystal structure of the complex of IgG1 Fc with FcγRIIIb reveals amino acids 234–238 to be important contact residues, and the subclass-specific variation in this area again can explain the lack of binding of IgG2 and IgG4 to this receptor (47, 110).

### FcRn

In the 1960s, the existence of a receptor responsible for the unusually long half-life of IgG (3 weeks, Table 1) and efficient transport from mother to young was first proposed by Brambell (164, 165). This was later confirmed by various groups and eventually cloned and identified as the neonatal FcRn (166–169).

Structurally, FcRn is strikingly similar to MHC-class I molecules (51, 52). Like MHC-class I and other MCH-class I-like molecules, FcRn is co-expressed with the non-glycosylated 12 kD β2-microglobulin, encoded on chromosome 15. The α-chain of human FcRn, a 45 kD polypeptide chain, is encoded on chromosome 19 at a locus harboring various other immune receptors (e.g., KIR, LAIR-1, CD89, CEACAM). Unlike FcRn from mice and rat, the human FcRn has only one potential glycosylation site (N102). It is located on the opposite face to the IgG-binding site and is also shared with that of all known FcRn sequences (mouse, rat, human being, macaque, pig, sheep, bovine, dromedary, and possum). FcRn does not bind its ligand at physiological pH (7.4). Only in the acidic environment of endocytic vacuoles (pH ≤ 6.5), where solvent exposed histidine residues in IgG are protonated, does binding to FcRn take place (169–171). Histidine residues within the Fc tail of IgG (CH2-CH3 interface) are critical for high affinity binding to residues within β2M and FcRn α-chain (51, 52, 151). H435 sits at the heart of this interface, and the lowered affinity of R435-containing allotypes of IgG3 to FcRn explains their shortened half-life and lowered placental transport (Table 1). Consequently, IgG3 has a normal half-life of 3 weeks and is transported efficiently across the placenta in individuals containing H435-containing IgG3 allotypes (g3m, 15, or 16) (20, 172).

That FcRn protects IgG from degradation has been confirmed by mouse models: IgG half-life is decreased in FcRn or β_2_-microglobulin deficient mice (166–169). Four times as much IgG is saved by FcRn-mediated recycling than is produced (173). While it was originally proposed that FcRn expression on endothelial cells is responsible for IgG recycling (174), later studies have shown that the strong FcRn expression on myeloid cells contributes equally to the half-life extension in mice (175). Likewise, overexpression of FcRn in transgenic animals results in higher IgG serum levels (176).

However, FcRn starts its function early in life by transport of IgG – and thereby humoral immunity – across the placenta from mother to young (168, 177–179) and in rodents also after birth by transport from mothers milk in the gut of suckling neonates. In rats, this FcRn expression is downregulated in the small intestines, which correlates with degradation of IgG in these cells (180).

In adult life, FcRn is expressed on many epithelial cells, and continues to function in IgG transport across FcRn expression epithelial barriers (181). FcRn is able (in all species) to bi-directionally transcytose cargo across polarized (both epithelial and endothelial) cells, but the net transport direction depends on the tissue (182–184). E.g., in syncytiotrophoblasts of the placenta, transport is directed away from the apical toward basolateral surfaces, while in the blood–brain barrier, it seems reversed (182–184) (abluminal to luminal or brain to blood); however, the involvement of FcRn in this transport is still debated (185).

Immunoglobulin G or IgG-antigen complexes have been described to be transported across mucosal surfaces, such as the intestinal cavity or respiratory epithelium, and thereby to function in immune surveillance (177, 181, 186, 187). With this role in mucosal immunity, it complements sIgA in immunoregulatory function as reviewed in Ref. (188).

As IgG can transport fully folded and functional proteins across epithelial barriers, this offers new possibilities for FcRn as an endogenous receptor to transport Fc-Fusion proteins or vaccine antigens across otherwise impermeable epithelial surfaces (181).

On mucosal cells, FcRn has been found to transport IgG and be involved in antigen sampling (177, 181, 187), and its expression on phagocytic cells (181, 189) has recently been found to enhance phagocytosis capacity of IgG-opsonized particles (190, 191). On antigen-presenting cells, this ingestion of IgG-complexes can lead to enhanced presentation (192–194). Similar to phagocytosis responses, the enhanced presentation likely requires the external sensing and cellular activation through FcγR and pattern-recognition receptors, handing the IgG-Antigen cargo over to FcRn at low pH (191, 192, 195). Thus, immunoglobulin activities including extended half-life, transport to young, and antigen sampling seems to be orchestrated through a single receptor, the MHC-class I-like FcRn. In contrast, other effector functions of IgG, such as phagocytosis and antigen presentation seem to be mediated by both FcRn and classical FcγRs.

## Alternative Receptors for IgG

### FcRL

Fc receptor-like proteins, consisting of six members (FCRL1-6) were originally identified as homologs of FcγR but were for a long time regarded as orphan receptors, mostly expressed on B-cells. Recently, however, FcRL4 and in particular FcRL5 were found to bind immunoglobulin as well, with the former recognizing IgA, IgG3, and IgG4, while FcRL5 binds all IgG subclasses similarly well, but not IgA. Both these receptors are expressed on B-cells, express an ITIM, and are known to downregulate B-cells after BCR cross linking through recruitment of SHP-1 (196, 197). Although FcRL5 seems broadly expressed on B-cells populations (198), FcRL4 is only expressed on subepithelial tissue B-cells (199) reportedly of mucosal origin, suggesting that perhaps this receptor is involved in negative feedback inhibition through antigen-specific IgG and IgA, respectively.

### TRIM21

Tripartite motif-containing protein 21 (TRIM21) is a cytosolic protein expressed in almost all cell types but highly expressed in immune cells. TRIM21 previously known as an autoantigen involved in several autoimmune diseases, e.g., systemic lupus erythematosus (SLE) (200). Later, TRIM21 was found to bind IgG with nanomolar affinity (53, 201). TRIM21 binds IgG in the Fc domain at the CH2–CH3 interface similar to FcRn and protein A/G, it competes for binding to IgG with protein A/G, and binding is independent of N-glycosylation of the CH2 domain (202). Later, it was demonstrated that TRIM21 functioned as an immunological sensor, targeting IgG-opsonized virus and bacteria for antibody-dependent intracellular neutralization by the ubiquitin-dependant proteosome (203–206). The detection of antibody opsonized virus by this receptor activates, requires proteasome and the ATPase and unfoldase VCP (206, 207). It also activates further signaling, and innate immunity responses are activated, characterized by the NF-kB, AP-1, and IRF pathways (208).

The unique localization of this receptor in the cytoplasm leaves many unanswered questions but simultaneously answering many. It helps to explain how partially opsonized pathogens may still be recognized and neutralized during the early phase of infection, escaping recognition by the complement and FcγR system. The relative importance of this system is still unknown during secondary infections, but may perhaps be relatively more important at locations where complement and the myeloid system are less prominently present, e.g., at mucosal surfaces of the gut.

### DC-SIGN

The cytotoxic activity of sialylated IgG has been described to be reduced in mice (125). Some of the immunomodulary activity of IVIg has been attributed to binding of sialylated fraction IgG to dendritic cell-specific ICAM-3 grabbing non-integrin (DC-SIGN or SIGN-R1 in mice) (209, 210). Since this glycoform of IgG represents only a small fraction (<10%) of all IgG in the blood and IVIG treatment typically requires very high doses, it was suggested that this fraction may be predominantly responsible for the immunomodulatory functions of IVIG. However, the precise mechanisms of this interaction are unknown, and still await confirmation – particularly in the human setting, but also in mice as some of the methods used to enrich IVIG for SA were found to predominantly – if not exclusively – enrich for Fab-associated SA (58, 59). It has been hypothesized that DC-SIGN binds to the CH2–CH3 interface of the Fc domain of IgG, owing to the opening up of the site, where DC-SIGN binds due to the charged sialic acid. The simultaneous closing the interaction site for FcγRs has then proposed to yield an anti-inflammatory IgG (50, 124). Crosslinking of SIGN-R1 in mice has been described to result in the release of IL-33, which in turn activates basophils to secrete IL-4, upregulating expression of the inhibitory FcγRIIb (209). However, recently an alternative receptor, the dendritic cell immunoreceptor (DCIR) has been put forward as an alternative candidate mediating the anti-inflammatory effect of silalylated-IgG, inducing upregulation of T regulatory cells, and minimizing Ig-complex-mediated airway hyperresponsiveness (211). To complicate things even further, a recent report claims sialic acid contents of IgG not to influence IgG binding to DC-SIGN, but to be rather Fab mediated (212).

Furthermore, a number of Siglecs (Siglec-2/CD22, Siglec-8, Siglec-9) have been implicated as ligands for IVIG (213–215), although in case of Siglec-9, there is evidence that lectin-specific antibodies in IVIG rather than sialylated antibodies are responsible for binding (216).

## Concluding Remarks

Immunoglobulin G-mediated responses diverge and depend largely on the type of secondary immune responses, which in turn depend on the type of antigen. This directs the immune response to a specific IgG subclass or subclasses – the function of which differs greatly between them. Besides this variation, the IgG profile of a given individual determined by their inherited allotypes can potentially influence the clinical manifestation of the immune response, which ultimately differs between individuals and populations. An even greater level of complexity is added by the profound variation seen in the glycosylation of the Fc tail, affecting binding to various receptors – the nature of which we are just beginning to understand.

## Conflict of Interest Statement

The authors declare that the research was conducted in the absence of any commercial or financial relationships that could be construed as a potential conflict of interest.
